# Revision Surgical Treatment of a Second Lumbar Ewing Sarcoma: A Report of a Rare Case

**DOI:** 10.1097/MD.0000000000001190

**Published:** 2015-07-31

**Authors:** Helin Feng, Jin Wang, Peng Guo, Jianfa Xu, Jiangang Feng

**Affiliations:** From the Department of Orthopedics, The Fourth Affiliated Hospital of Hebei Medical University, Shijiazhuang, Hebei, P. R. China.

## Abstract

We report a case of a 58-year-old man who presented initially with lumbar pain.

According to radiography, computed tomography, magnetic resonance imaging, and bone biopsy results, Ewing sarcoma (ES) was diagnosed. Tumor resection was performed, followed by chemotherapy and radiotherapy; pathology confirmed the diagnosis of ES. After surgery, the tumor recurred twice with progressive symptoms, meriting repeated excisional surgery. At the 4-year follow-up, the patient showed apparent improvement, with return of function and strength and resolution of pain.

We discuss its clinical features and treatment in the light of the current knowledge.

## INTRODUCTION

Ewing sarcoma is the second most common malignant bone tumor in children and adolescents.^1^ It most commonly occurs in the long bones of the extremities (predominantly the femur) and the pelvis,^2^ and rarely occurs in the spine.^3^ Surgery is currently the most effective treatment to decompress the spine and achieve the maximum possible resection.^4^ Spinal ES tumors are difficult to remove en bloc as they entrap the vertebral body.^5^ Furthermore, they are associated with high rates of recurrence and metastasis. Here, we report an unusual case of a second lumbar (L2) ES, in which 3 surgeries were performed because of local recurrence. Surgical treatment yielded satisfactory results, with no involvement of other sites or organs.

## CASE REPORT

**FIGURE 1:**
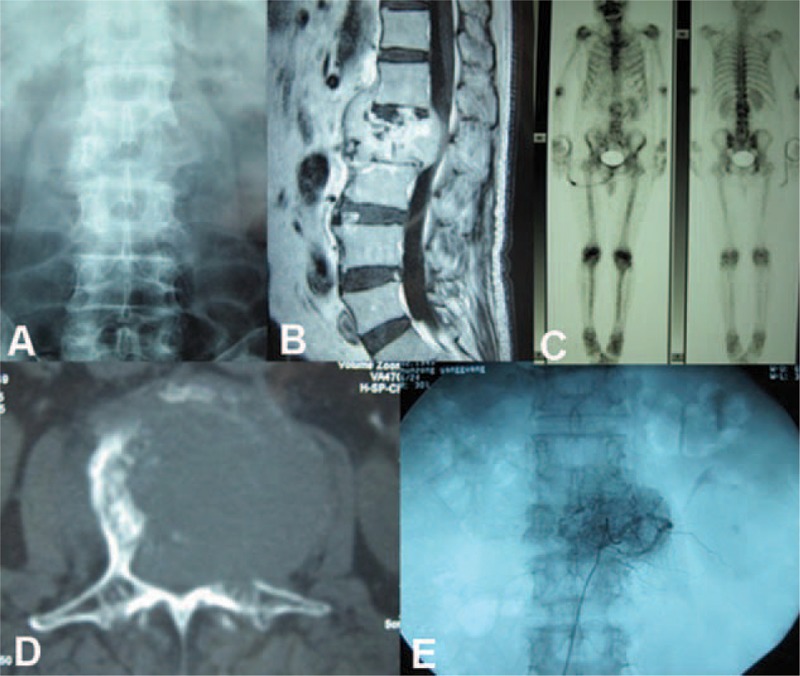
(A) Preoperative radiography. (B) MRI. (C) Bone scintigraphy. (D) CT. (E) Preoperative vertebral tumor embolization.

**FIGURE 2:**
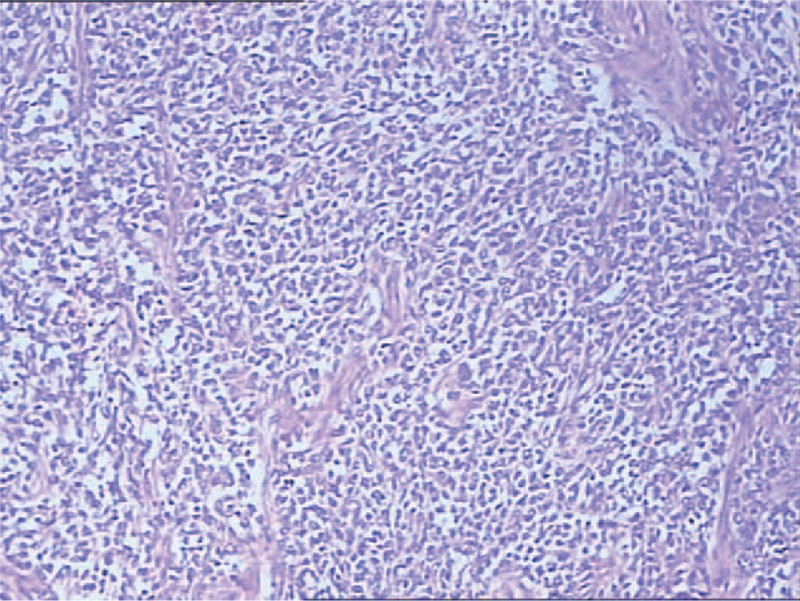
Hematoxylin and eosin stained sections of tumor tissue showing the classic histologic appearance of a small blue cell neoplasm with nests of small round tumor cells with scanty cytoplasm.

**FIGURE 3:**
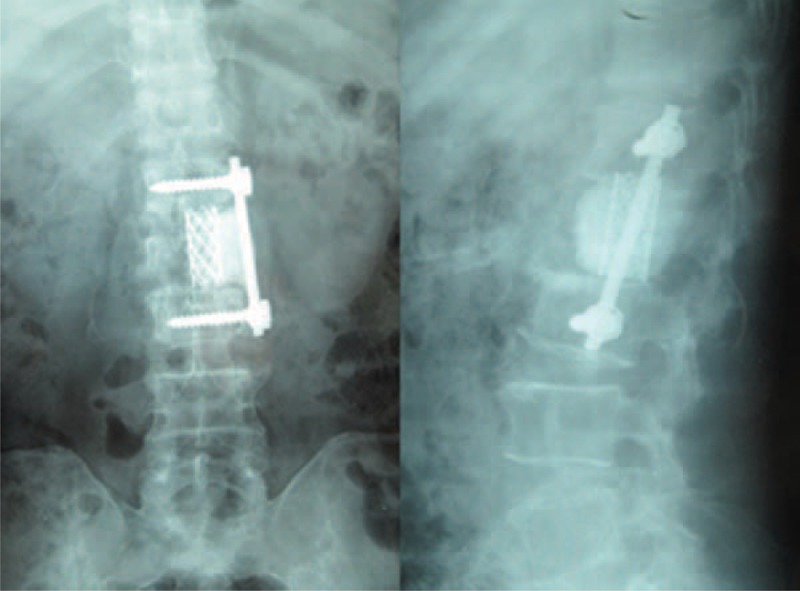
Postoperative radiography. The vertebra was fixed with titanium mesh and a single pedicle screw and was padded with bone cement.

The patient was a 58-year-old man who had experienced low back pain for 3 months. He had been diagnosed and treated for lumbar intercalated disc herniation at other hospitals. However, his symptoms had worsened, with a decrease in lower limb muscle strength and neurogenic bladder and bowel dysfunction. The patient was transferred to our hospital and examined with radiography, computed tomography (CT), magnetic resonance imaging (MRI), 99mTc-methylene diphosphonate bone scan (Figure 1), and bone biopsy (Figure 2). According to the results of these tests, ES occupying the second lumbar vertebra with paraplegia was diagnosed. The first surgery was performed on July 8, 2005. Vertebral tumor blood vessel embolism was performed preoperatively, and the left centrum vertebra was found to be in poor condition. An improved kidney incision was made, and the vertebra was fixed with titanium mesh and a single pedicle screw and padded with bone cement (Figure 3). The patient was transfused with 2800 mL of blood. The patient was treated with a neurotrophic drug postoperatively, and pathology confirmed the diagnosis of ES. The patient recovered within 6 weeks. Systemic chemotherapy and radiation were administered after the surgery.

**FIGURE 4:**
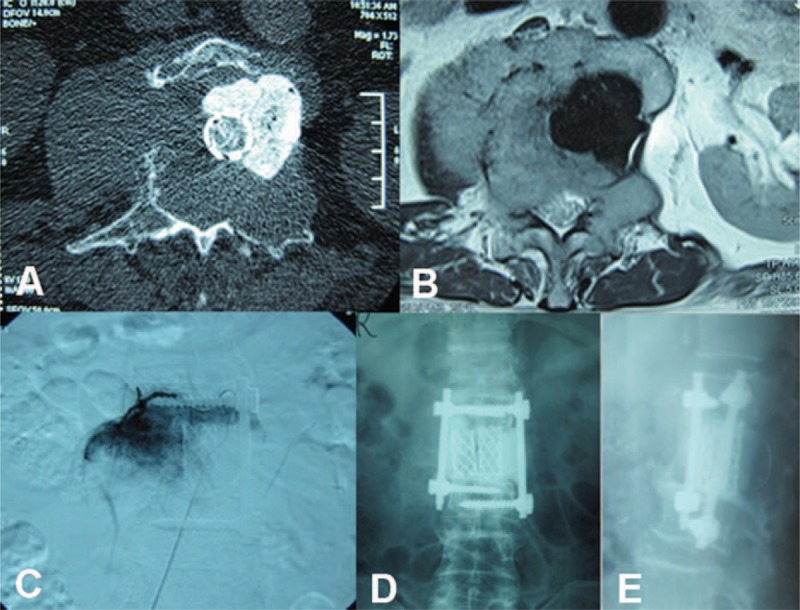
(A, B) Second preoperative computed tomography and magnetic resonance imaging showing recurrence of the tumor on the right side of the spine. (C) Preoperative vertebral tumor embolization. (D, E) Postoperative radiograph showing the titanium mesh and single pedicle screw.

The patient visited the hospital again on August 8, 2008, because of progressively worsening pain in his waist and myasthenia of his limbs for 4 weeks. The results of CT and MRI (Figure 4A and B) showed local tumor recurrence limited to the right side of his vertebra. Accordingly, we performed a second surgery. Tumor blood vessel embolism was performed preoperatively (Figure 4C), and the recurrent tumor, measuring 5 × 5 × 4 cm, was excised. The tumor was only partly excised because it had invaded the psoas major muscle. We used titanium mesh and a single pedicle screw to fix the vertebra, and bone cement to enhance the connection (Figure 4D and E). The patient was transfused with 2600 mL of blood.

**FIGURE 5:**
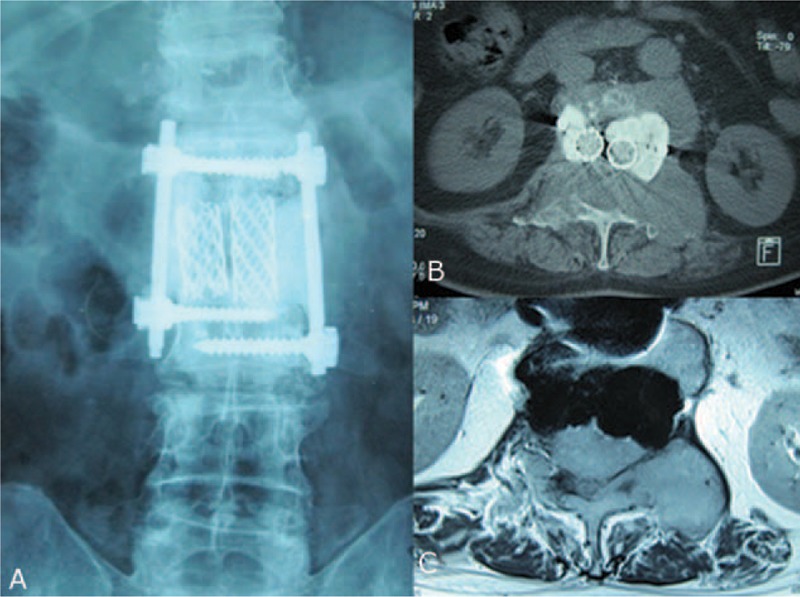
(A) Third preoperative radiography, (B) computed tomography, and (C) magnetic resonance imaging showing tumor recurrence on the left part of the pedicle.

**FIGURE 6:**
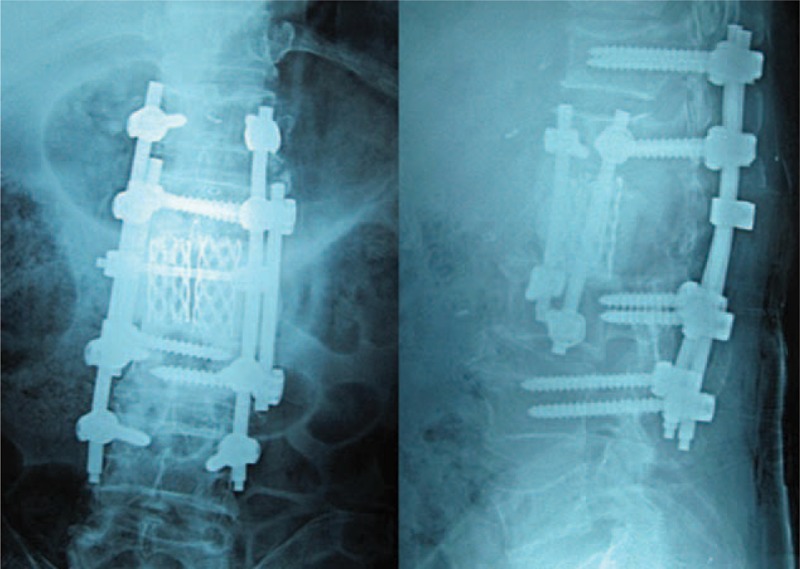
Postoperative radiography showing the posterior screw rod fixation.

On February 25, 2010, the patient could not easily move both of his lower extremities because of dysuric syndrome. On physical examination, muscle strength of the right lower limb was scored as 2 and that of the left was scored as 1. Results of other examinations showed that the tumor had recurred locally, mainly around the pedicle (Figure 5). A third surgery was performed to decompress the posterior vertebra and excise the tumor. Lumbar posterior fixation was then performed. Considering the bolts in the front of the first and third centrum, we chose a shorter pedicle screw to fix the vertebra (Figure 6). The surgery was successful, although the patient lost 1000 mL of blood and was transfused with 700 mL of blood. After 4 weeks, the patient experienced bilateral lower limb recovery and was able to walk with a supporting apparatus. The patient was alive as of September 25, 2014, and his bodily functions had recovered well. The patient provided written informed consent for the publication of these case details, and the consent procedure was approved by the Human Ethics and Research Ethics committees of the Fourth Hospital of Hebei Medical University, Hebei, China.

## DISCUSSION

The spine is frequently involved in cases of metastatic ES, but primary involvement of the spine is much less frequent.^6^ ES is thought to arise from neural crest cells.^7^ MRI^8^ plays a predominant role in evaluating the features of the lesion and developing a preoperative surgical plan for the ES family of tumors. The signal intensity of spinal peripheral ES is usually homogeneous and is enhanced homogeneously after the administration of contrast agents. Spinal peripheral ES tends to involve paraspinal tissue, and spinal metastases can pathologically cause further paraplegia.^9^ Thus, we should consider surgical therapy when treating patients with spinal ES in order to improve quality of life by providing neurological improvement and spinal stabilization.^10^ Surgery and fixation are also good treatments to relieve the pain caused by spinal cord nerve root compression and increase spinal stability. After spinal ES resection, spinal stability is compromised to different degrees and must be restored by internal fixation to effectively protect the spinal cord. A clear diagnosis can be helpful for choosing the best treatment and preserving functionality for paraplegics, particularly as every minute counts.

Surgical ablation is a common and effective treatment for primary spinal tumors, especially those with local invasion and metastases.^11^ Owing to the complex anatomical structure of the spine and insufficient surgical resection, recurrence is difficult to avoid. It is also difficult to abide by the principle of Enneking while removing the tumor as completely as possible and protecting the nearby nerves and major blood vessels. Assuming the patient's general condition allows for elective surgery, revision surgery is necessary for some patients to avoid the occurrence of paraplegia and prolong survival time.
